# Enhancing Consumer Online Purchase Intention Through Gamification in China: Perspective of Cognitive Evaluation Theory

**DOI:** 10.3389/fpsyg.2020.581200

**Published:** 2020-11-23

**Authors:** Yan Xu, Zhong Chen, Michael Yao-Ping Peng, Muhammad Khalid Anser

**Affiliations:** ^1^Business School, Yango University, Fuzhou, China; ^2^School of Economics, Fujian Normal University, Fuzhou, China; ^3^School of Economics and Management, Foshan University, Foshan, China; ^4^School of Public Administration, Xi’an University of Architecture and Technology, Xi’an, China

**Keywords:** online shopping, gamification, cognitive evaluation theory, game dynamics, consumer enjoyment

## Abstract

The application of game elements of gamification in online shopping is attracting interest from researchers and practitioners. However, it remains unclear how gamification affects and improves consumer purchase intention on online shopping platforms, which still leaves a gap in our knowledge. To narrow this theoretical gap, a theoretical model has been built in this study. This model adopts cognitive evaluation theory to explain the impact of gamification elements on consumer purchase intention. Data was collected from 322 online shopping consumers who used a flash game to test their purchase intention after playing games. The results show that game rewards, absorption and autonomy of gamification positively enhance sense of enjoyment, and that it helps people meet their psychological needs, which ultimately affects the online purchase intention of consumers. This study is helpful in analyzing the factors involved in the successful introduction of gamification on online shopping platforms in more detail.

## Introduction

As mobile applications and social media have evolved, competition in the online shopping market has grown fiercer, with many businesses working to affect consumer behavior (Wang and Fesenmaier, 2003). An increasing number of businesses are competing for a share of the market by attracting active consumers. As a relatively new paradigm for engaging people, gamification is applied as a strategy to influence and motivate people to participate in education, marketing, training, networking, and health-related activities (Bunchball, 2010). Gamification is the implementation of dynamic components and elements of games (Zichermann and Linder, 2010; Mullins and Sabherwal, 2020) that are not directly related to games (Bunchball, 2010) and appear in non-game contexts (Deterding et al., 2011). The term “gamification” was first used in 2002, but it was not until 2010 that this concept of gamification became popular (Mitchell et al., 2020).

Introducing game mechanics into business is the science of enriching consumer interaction, while games for commercial purposes are still under development (Zichermann and Cunningham, 2011). In this sense, it is urgent for platforms to learn how to introduce game mechanisms into their business to provide their consumers with a rewarding, enjoyable, and fun experience. As an emerging way to attract consumers, gamification is being used in marketing, school education and training, on the Internet, and in related industries (Huang and Cappel, 2005; Silverman, 2011; Hordemann and Chao, 2012; Kankanhalli et al., 2012). In this context, the design of game elements, such as inspiration, competition mechanisms, and shock, is used to increase the value of high enjoyment to attract consumers (Simões et al., 2013; Seaborn and Fels, 2015; Müller-Stewens et al., 2017; Mullins and Sabherwal, 2020).

Taking this context into account, gamification has been clearly deemed as a means of driving consumer behavior. Gamification is the utilization of game design elements in non-game contexts (Deterding et al., 2011; Mitchell et al., 2020). Since 2002, gamification (Hamari and Lehdonvirta, 2010; Deterding et al., 2011) and persuasive technologies (Fogg, 2002) have been harnessed for business purposes and to influence customer behavior. The control of game elements in gamification may have a positive impact on the experience of playing games and the generation of customers’ intention (Poncin et al., 2017; Mitchell et al., 2020; Mullins and Sabherwal, 2020). For instance, Alibaba has set up a game mechanism on its payment platform, on which the quantity of trees planted depends on individual walks, so as to fulfill its social responsibility and stimulate consumption through the platform. On the other hand, as gamification is heavily driven by information communication technologies, it is natural to address interrelations between gamification and online behavior of consumers (Huang et al., 2017). For example, JD.com, a large online shopping platform in China, enables people to gain points, known as beans, when they make purchases; these beans can then be exchanged for other commodities or planted on the game platform in order to obtain more beans and increase consumer willingness on this platform.

Although there has been a lot of research on online consumer behavior (Chen et al., 2015), there is a lack of research on gamification from the perspective of consumer behavior (Sigala, 2015). In the context of fierce competition among online shopping platforms, many such platforms not only face domestic competitors, but also have to consolidate the barriers to entry of foreign competitors (Xi and Hamari, 2020). Thus, the concept of gamification is an important source of stimulation in the marketing theory of consumer behavior decision (Tobon et al., 2020; Xi and Hamari, 2020), and it provides specific directions for researchers in the study of online marketing. Therefore, this study aims to explore the effect of gamification on consumers’ online purchase intention.

For this purpose, a theoretical model has been developed to predict the impact of consumers’ enjoyment in the game on their purchase intention by drawing on cognitive evaluation theory (CET) (Ryan and Deci, 2000a, b; Deci and Ryan, 2010; Mitchell et al., 2020). According to CET, when people are involved in certain activities, they have psychological needs such as autonomy and absorption. When individuals feel that their demands need to be met, they will trigger intrinsic motivation and feel a greater sense of enjoyment, which, in turn, will lead to more engagement in activities (Lee and Yang, 2011) and ultimately affect consumer behavior. Since the main purpose of gamification is to develop willpower and high-quality forms of motivation, CET helps us understand the changes in consumer behavior in the context of gamification. Based on CET, a model has been developed and tested in this study to explore how game elements affect users’ psychological needs and increase consumers’ sense of enjoyment, thereby influencing their purchase intention.

According to the literature on meaning (Webster and Ahuja, 2006; Sen et al., 2008; Seaborn and Fels, 2015), people derive meaning when their activities are consistent with core aspects of enjoyment. Autonomy, rewards, and absorption are important factors for the success of gamification (Sigala, 2015; Mitchell et al., 2020), as well as lying at the core of CET. According to the above explanations, this study intends to propose relevant research contributions on the basis of the following theoretical gaps: (1) applying CET to explore the important role of gamification in consumer online purchase intention; (2) focusing on verifying characterized game elements of gamification, which is conducive to filling the gap of variable measurement in the theoretical literature on CET; (3) enriching applications of gamification for business and academics, particularly those that add new features and gameplay mechanics (Huotari and Hamari, 2017) to ensure both customer enjoyment and the success of business objectives.

## Literature Review and Theory Development

### Cognitive Evaluation Theory

Cognitive evaluation theory is a psychological theory that aims to explain the effect of extrinsic results on intrinsic motivation. CET proposes the concept of “intrinsic incentive,” which is also known as “intrinsic motivation.” The theory suggests that people are more likely to participate in an activity when they have intrinsic motivations such as an experience of enjoyment (Agarwal and Karahanna, 2000; Gottschalg and Zollo, 2007; Beecham et al., 2008). Deci and Ryan (2000) proposed three types of motivation: extrinsic regulation, intrinsic regulation, and intrinsic motivation. Their study emphasized that motivation needs to be intrinsic rather than extrinsic. The central focus of Deci and Ryan’s research was on intrinsic motivation and the antecedents that increase persistence. They defined intrinsic motivation as performing an activity solely for inherent satisfaction. This is a broader view that people motivated intrinsically are more stimulated and perform better than others (Cerasoli et al., 2014). Although researchers regard intrinsic motivation as an inherent quality, the maintenance and enhancement of this motivation depends on the social and environmental conditions around the individual. Deci and Ryan’s CET proposed that individuals’ significant psychological needs are satisfied when the individuals perceive that they can regulate their behaviors. Intrinsic motivation is supported by social and environmental factors, such as events and conditions, that enhance an individual’s sense of autonomy and competence, whereas it is undermined by factors that diminish perceived autonomy or competence (Deci and Ryan, 1980; Chae et al., 2017). Withdrawing on theoretical foundation, this study adopts CET to build conceptual framework of gamification and expands upon how gamification elements are key determinants of consumer enjoyment, intrinsically motivated purchase intention.

By extending such aspects of CET to this study, it is possible to consider the behaviors of extrinsic regulation to be motivated by external factors such as awards and competition, and the behaviors of intrinsic regulation to be motivated by internal factors such as absorption and autonomy. When an individual realizes that the causation originates from the behaviors mentioned above, intrinsic motivation appears. An example of intrinsic motivation is enjoyment. When people are dominated by intrinsic motivation, they will stick to a task for longer and like it more (Deci, 1985). The contribution of CET is that it proposes the factors that enable people to generate intrinsic motivation, which are specifically autonomy and competence. Autonomy means the willpower or willingness to do a task; competence refers to the feeling of being effective (Silverman, 2011; Santhanam et al., 2016; Huang et al., 2017), such as getting rewards, being addicted to games, and participating in competition.

### Consumer Enjoyment

Consumer enjoyment is “a necessary response of humans to activities with computers as intermediaries” (Laurel, 2013). When consumers are attracted by a game, a sense of enjoyment will be generated (Jacques, 1995). Intrinsic motivation of expected enjoyment derives from the pleasure or inherent interest in doing something (Gagné and Deci, 2005). Curiosity, fun, or enjoyment can all be intrinsic motivations (Kim and Drumwright, 2016). Based on CET, intrinsic motivation derives from one’s preference for an activity. People will gain inherent satisfaction from doing it, intrinsic motivation reflects the desire to engage in a task for its enjoyment (Tao and Yun, 2019). Enjoyment of an activity is generally viewed as an important intrinsic motivation (Kim and Drumwright, 2016; Hew et al., 2018). Consumer enjoyment is important because it allows people to have a positive outlook on human–computer interaction, thus increasing future motivation for repeated interactions with games (Kim and Moon, 1998; Webster and Ahuja, 2006). This, in turn, leads to the success of a game (Hwang and Thorn, 1999).

Studies have shown that consumer enjoyment can develop positive attitudes through certain activities, such as gaining rewards, absorption in games, participation in competition, and feeling self-control (Schaufeli et al., 2002). These subdimensions represent the emotional, cognitive, and physical aspects of consumer enjoyment (Chen et al., 2015). In this study, autonomy is defined as the voluntary participation of a consumer in an activity designed by gamification and the consumer’s continuous efforts to gain rewards in the face of difficulties. Competition comprises the senses of meaning, pride, and challenge, as well as the inspiration and passion of consumers. Enjoyment refers to the extent to which a consumer’s experience culminates in pleasure and excitement triggered by the online gamified environment. Some scholars hold that some psychological needs should be satisfied if people want to keep their intrinsic motivation (i.e., enjoyment) (Ryan et al., 2006). In other words, when a person’s basic psychological needs of competence, autonomy, and relevance are satisfied by an activity, greater enjoyment will be gained.

In this case, enjoyment is the extent to which an individual obtains a pleasant experience while playing games (Huotari and Hamari, 2017). CET predicts that if people consider an activity involving a certain form of technology to be enjoyable, the intrinsic motivation will be increased and extrinsic behaviors will ultimately be affected (O’Brien, 2010; Lee and Yang, 2011). In the field of online shopping, enjoyment is considered to be a motivational state that can influence the degree and focus of consumption (Bunchball, 2010). Purchase intention is defined as a spontaneous and powerful shopping tendency and a shopping process that is dominated by consumers themselves (Rook and Fisher, 1995). In a state of enjoyment, consumers tend to feel environmental stimuli and arousal impulses (Wang and Li, 2016). As the purpose of gamification is mainly to make consumers’ activities more enjoyable (Bunchball, 2010), enjoyment is a significant intrinsic motivation that determines whether consumers participate in designed gamified shopping environment and affects purchase intention. On this basis, we propose the following hypothesis:

H1: Consumer enjoyment has a positive impact on online purchase intention.

### Gamification

Gamification can collect user data for salespeople to observe user preference (Nelson, 2005). If users develop a negative attitude toward the instrumental trait of a certain game, they will not play the game anymore, which hinders the development of a favorable brand attitude and game skills (Kwak et al., 2012; Xi and Hamari, 2020). Some scholars have suggested conducting a survey on specified gamification design elements, so as to improve the design and obtain the benefits of gamification (Kim and Johnson, 2016; Mitchell et al., 2020; Mullins and Sabherwal, 2020). Thus, it is quite important to analyze the use of gamification business applications to understand the impact of gamification and social cognition on e-commerce success (Wakefield et al., 2011; Tobon et al., 2020; Xi and Hamari, 2020). According to CET, increasing all aspects of value can enhance more customers’ experience of enjoyment and ultimately promote online consumer behavior.

Gamification can serve to enhance consumer enjoyment with online shopping (Huotari and Hamari, 2017). The factors that stimulate consumer online shopping are closely associated with the motivation to participate in games and can be divided into two categories: intrinsic motivation and extrinsic motivation. Both extrinsic and intrinsic motivation play a significant role in online shopping. However, according to CET, intrinsic motivation represents enjoyment in an activity for its own sake (Mekler et al., 2017). For example, people who shop online because they enjoy looking over new things and expanding their consumer knowledge are intrinsically motivated to be there. However, some scholars agree that the intrinsic motivation factor is more important than extrinsic motivation and has a greater impact on consumer behavior (Reiss, 2004; Deterding et al., 2011; Tobon et al., 2020).

Moreover, merely adding gamification mechanics such as challenge and fantasy in a smart interface is not enough to significantly enhance the quality of the perceived experience (Insley and Nunan, 2014; Mitchell et al., 2020). The purpose of gamification is to increase consumer motivation and facilitate consumers’ participation in gamification activities through intrinsic and extrinsic motivators, and to provide a pleasant experience (Von Ahn and Dabbish, 2008; Conaway and Garay, 2014; Xi and Hamari, 2020). Reward, competition, autonomy, and absorption are the most common game dynamics in the literature on gamification (Agarwal and Karahanna, 2000; Gottschalg and Zollo, 2007; Liu et al., 2007; Hordemann and Chao, 2012; Kapp, 2012; Mullins and Sabherwal, 2020), these elements must be available in order for gamification to be used (Conaway and Garay, 2014). As a result, consumers are encouraged to further participate in the system (Gottschalg and Zollo, 2007), which ultimately affects purchase intention. Furthermore, a gamified campaign needs to be well executed in order to achieve the intended goals (Lucassen and Jansen, 2014). To represent components of gamification specifically, reward, competition, autonomy, and absorption have been adopted as measurement dimensions of gamification in this study (Agarwal and Karahanna, 2000; Gottschalg and Zollo, 2007; Liu et al., 2007; Hordemann and Chao, 2012; Kapp, 2012), and Table 1 summarizes the definitions of these dimensions.

**TABLE 1:** Definitions of variables in gamification.

Game dynamics	Game elements	Description
Rewards	Points, levels, virtual gifts	Consumers earn points as a reward by completing pre-assigned tasks. Points are a game element of gamification, which induces consumers to strive for more rewards. Levels create a dynamic that encourages consumers to make efforts to improve their status through achieving predefined goals or reaching milestones of gamification. Emblems or loots indicate the valuable activities of a person, thus motivating players to obtain tangible rewards and then show their achievements (Reiss, 2004; Gagné and Deci, 2005; Gottschalg and Zollo, 2007; Hordemann and Chao, 2012).
Competition	Points, levels, leaderboard	Leaderboard offers consumers the opportunity to compare and compete with others. Consumers attempt to get more points in an activity, reach a higher level, and earn more emblems and loots (Gottschalg and Zollo, 2007; Liu et al., 2007).
Autonomy	Decision, judgment, sharing behavior	Autonomy defines the extent to which an individual can control and determine the consequences of his/her behaviors. In general, human beings fight for as much autonomy as possible. Competence refers to having goals and relevant skills to achieve them (Deci and Ryan, 2000; Ryan and Deci, 2000a; Kapp, 2012). Autonomy can be realized by allowing users to choose their own tools and to self-assign tasks (Beecham et al., 2008; Schell, 2019). Consumers’ perceived autonomy is evoked by the participation in gamification.
Absorption	Spending time, control	Consumers indulge in the process of gamification and even forget themselves. A typical example is the consumer’s emotion towards a game when he/she is deeply involved in the game (Agarwal and Karahanna, 2000).

Based on CET, researchers hold that consumer competence is an important prerequisite for triggering enjoyment. When a consumer feels that he/she is controlled or forced to do something (e.g., participate in an unpleasant competitive relationship), any external condition will reduce the intrinsic motivation and lessen the experience of enjoyment (Antin and Churchill, 2011). Players’ voluntary enjoyment is the key element of a game (Huotari and Hamari, 2017).

Playing a game means the player is in an environment where he/she has autonomy (Gagné and Deci, 2005), and people participate in the game of their own free will. This is an exact reflection of autonomy. Game activities, such as completing tasks, defeating other players, and developing strategies to achieve goals with other players, can help people meet their psychological needs of autonomy, competence, and relevance (Deci and Ryan, 2000; Beecham et al., 2008; Mitchell et al., 2020), and improve the inner experience of enjoyment. According to CET, people have more fun when engaging in activities in which they are interested or in activities that can reflect their personal value (Ryan et al., 2006). When external conditions are able to meet internal psychological needs, external factors can increase the intrinsic motivation and enable people to experience enjoyment (Antin and Churchill, 2011; Mitchell et al., 2020). In other words, the greater the freedom perceived by consumers when making orders on an online shopping platform, the greater the efficiency in triggering the consumers’ intrinsic motivation to engage in the consumption process and in further satisfying their psychological needs (Rogers, 2017). From this logic, we can infer that when gamification is applied in the context of online shopping, enjoyment can be more easily triggered if the need for autonomy is satisfied. Based on the above discussion, we have developed the following hypothesis:

H2: Autonomy of gamification has a positive impact on enjoyment.

In the design of gamification, rewards are what the user receives as a return for completing pre-assigned tasks. Rewards and challenges have been identified as the two mechanisms that are most commonly used for gamification (Tobon et al., 2020). Rewards can motivate consumers to make every effort to improve their level and get more points or loots (Deterding et al., 2011). CET confirms the importance of rewards. Players can earn points, rise to a higher level, or get badges or discounts as rewards (Hofacker et al., 2016). People are motivated to gain more rewards. For example, the ranking place on the leaderboard can stimulate a player’s desire to compete with others for better scores (Hordemann and Chao, 2012). These reward mechanisms are helpful in intensifying the intrinsic motivation to get a better experience of enjoyment (Przybylski et al., 2010). Thus, by helping people to meet their psychological needs, rewards can stimulate people’s intrinsic motivation to get a better experience of enjoyment from specific activities.

According to CET, obtaining real returns through gamification can enhance the consumer experience and help consumers achieve higher satisfaction (Deci and Ryan, 2000). Moreover, some scholars believe that rewards can bring a higher level of enjoyment (Johnson et al., 2018). Through the continuous accumulation of points, consumers have confidence in their own capability, which can then improve their sense of enjoyment (Francisco-Aparicio et al., 2013). Consumers can also exchange points earned from rewards with virtual discounts or products according to their own needs. For example, consumers are rewarded for reaching higher levels, which gives them a sense of achievement and allows them to feel self-worth. Hence, the more rewards consumers gain through gamification, the more they consider themselves valuable (Przybylski et al., 2010) and the easier it is to generate enjoyment. Thus, we propose our third hypothesis:

H3: Rewards of gamification have a positive impact on the generation of enjoyment.

Absorption in gamification has a strong influence on individual behavior change (Silic and Lowry, 2020). According to CET, people can count on intrinsic motivation to generate stable actions when they are immersed in their own world (Rook and Fisher, 1995). For consumers using gamification, absorption is a state of enjoyment. Under this state, players can be absorbed in these games. This can be seen as a process of high enjoyment. Gamification allows players to immerse themselves in a virtual world, helping them escape from some of the problems in the real world. Some players may be absorbed in a game, enjoy mental relaxation, and feel that time passes faster than usual. Some scholars call this state a “flow state,” under which people may only be aware of activities they participate in, or of the specific environment they are in Mauri et al. (2011). Some scholars believe that games can improve and regulate emotions, and that participants experience higher absorption after completing game tasks, thus generating more positive emotions (Yang et al., 2020) and stimulating more powerful motivations (Silic and Lowry, 2020). Therefore, consumers’ absorption in a game may have a positive influence on their enjoyment. Players who are obsessed with a game may have more enjoyment intentions. Therefore, we have developed our fourth hypothesis as follows:

H4: Absorption of gamification has a positive impact on the generation of enjoyment.

Gamification by nature thrives in the context of competition to win (Morschheuser et al., 2016; Mitchell et al., 2020). People challenge each other to achieve the best results. Leaderboards can show game results and celebrate the winners. The basic property of games, no matter whether they are multi-player games, single-player games, or other single-user experiences, is to compete for a specific goal. When participants need to present themselves as active solutions on a competitive platform, they will actively pay more attention to participating in the gamified environment (Deng et al., 2016). Consumers immerse themselves in games through the competitive environment designed by gamification. The satisfaction arising from competition with others is able to enhance the consumer’s intrinsic motivation and enjoyment of online shopping. This is because people get satisfaction from comparing themselves with others. The literature on CET indicates that individuals are motivated to achieve better results in competition (Ryan and Deci, 2000a, b) and to obtain a better experience of enjoyment. Therefore, we propose the following hypothesis:

H5: Competition of gamification has a positive impact on the generation of enjoyment.

**FIGURE 1:**
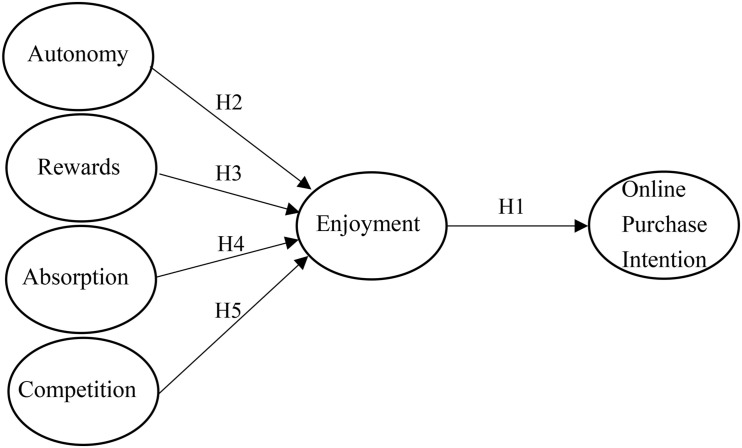
Research model.

This study extends CET by identifying the antecedents of need satisfaction, and it develops a research model to explain consumer enjoyment with gamification, as shown in Figure 1.

## Methodology

### Sampling

Taobao, China’s largest online shopping website, has 576 million users. In November 2019, Taobao launched a game called “Stackopolis.” In this game, consumers can get rewards or discounts, and a large number of consumers have played the game. We adopted a questionnaire survey to test our hypotheses. Given that our model covers different constructs, such as consumer absorption in games and self-control, we used a structural equation model to discover through path analysis whether the relationship between these variables is statistically significant (Deci, 1985). The method used to develop measurement items and collect data is discussed in more detail in this section.

The data was collected from Chinese consumers who shopped on Taobao in November 2019. We conducted a survey using purposive sampling. Taobao was selected as the subject of the case study because as an online shopping website it is second only to Amazon in the world, which means that it allows for sufficiently representative sampling required to discuss the impact of gamification on consumer purchase intention. To improve the response rate, we offered each participant RMB 20 once they had completed the questionnaire. A total of 350 questionnaires were collected. After the questionnaires were checked, 28 questionnaires were omitted as invalid. The number of valid questionnaires was 322. The main targets for data collection were consumers between the ages of 20 and 40, as they are the biggest consumer groups in the online shopping market. The information about the sample profile is shown in Table 2.

**TABLE 2:** Descriptive statistics.

Characteristic		Scale (%)
Gender	Male	146 (45.3%)
	Female	176 (54.7%)
Age	20-29	113 (35%)
	30-39	78 (24.2%)
	40-49	71 (22.1%)
	≥50	60 (18.7%)
Education level (completed)	High school or below	59 (18.2%)
	College	171 (53.2%)
	Graduate school or above	92 (28.6%)
Occupation	Public servant	31 (9.6%)
	Manufacturing	20 (6.2%)
	Business	93 (28.9)
	Professional	19 (5.9%)
	Unemployed (e.g., student, retired, housewife)	159 (49.4%)
Total		322

When self-report questionnaires are used to collect data at the same time from the same participants, common method variance (CMV) may be a concern. A *post hoc* Harman one-factor analysis was used to test for common method variance (Podsakoff and Organ, 1986). The explained variance in one factor is 38.54%, which is smaller than the recommended threshold of 50%. Therefore, CMB is not problematic in this study (Harman, 1976).

### Procedure

This is a cross-sectional study whose research framework and survey instrument have been approved by the Institutional Review Board of National Kaohsiung University of Science and Technology. The researchers contacted the consumers who were willing to receive the questionnaire by email first. Each survey package contained a covering letter explaining the purpose of the survey and the survey instrument. Before filling out the questionnaires, consumers were asked to understand the right of attending survey to ensure research ethical aspects.

### Instrument

A questionnaire survey was used to collect data and develop measurement items using a five-point Likert-type scale, in which “1” means “strongly disagree” and “5” means “strongly agree.” The English questionnaire was translated into Chinese by a researcher whose first language is Chinese, and the Chinese questionnaire was translated into English by another researcher to ensure that the meaning of items did not change because of translation. Afterward, the questionnaire was sent to six consumers who had experience in bilingual online shopping to further check the accuracy of the translation and the clarity of the questionnaire, and then some expressions were adjusted on the basis of their feedback.

Items of enjoyment were adopted from Sykes et al. (2009) to Kim et al. (2013), and we adopted items for autonomy from Sheldon et al. (2001) to Jang et al. (2009). Items for rewards were adopted from Kankanhalli et al. (2005); Sen et al. (2008), O’Brien (2010); Wakefield et al. (2011). Items for competition were adopted from Chen et al. (1998); Ma and Agarwal (2007), Lee and Yang (2011), and items for absorption from Schaufeli et al. (2002). Finally, we adopted items for online purchase intention from Huang et al. (2017). In the scale of purchase intention, VIP service can be referred as offering consumers a very individual form of online shopping. We collected the data by means of a questionnaire (see Table A1).

### Data Analysis Strategy

The hypotheses of research framework have been tested and paths have been included via structural equation modeling in this study. Measurement model was performed using IBM-SPSS 25 and SmartPLS 3.0 statistical program; Partial least squares structural equation modeling (PLS-SEM) was adopted to construct the structural model, specifically, verification of the structural model was performed using SmartPLS 3.0 (path analysis).

## Results and Analysis

### Measurement Model

A two-stage analytical procedure was used for the data analysis (Deterding et al., 2011). The measurement model for reliability and validity was assessed in the first stage, and the structural model was examined in the second stage to test the hypotheses (Hair et al., 1998).

Confirmatory factor analysis (CFA) for latent variables of Smart-PLS 3.0 and SPSS 25 were used as the analytical tools for this study. All factors have strong significance, so the intrinsic consistency and convergent validity of each scale are supported, indicating that the structure is sufficiently reliable (Hair et al., 1998; Table 3).

**TABLE 3:** Validity and correlation of constructs.

	α	CR	AVE	1	2	3	4	5	6
(1) Absorption	0.906	0.934	0.779	**0.883**					
(2) Autonomy	0.839	0.902	0.754	0.008**	**0.868**				
(3) Competition	0.875	0.907	0.709	0.487**	0.095**	0.842			
(4) Enjoyment	0.849	0.909	0.770	0.522**	0.267**	0.476**	**0.878**		
(5) Purchase intention	0.836	0.886	0.663	0.413**	0.298**	0.498**	0.455**	**0.814**	
(6) Rewards	0.928	0.949	0.822	0.351**	0.167**	0.678**	0.416**	0.529**	**0.907**

α = Cronbach’s alpha; (1) Square root of AVE for each latent construct is given in diagonals. (2) AVE, Average variance extracted; CR, Composite reliability. **if p < 0.01. Bold value represent square root of AVE for each latent construct is given in diagonals.

We have examined the average variance extracted (AVE) in order to assess discriminant validity. If the AVE from a construct is greater than the variance shared between the construct and the other constructs in the model, a satisfactory discriminant validity is obtained (Chin, 1998). The square root of the AVE of each construct should exceed its correlation with all the other constructs. It can be seen from Table 3 that the AVE for each construct is larger than its correlation with all the other constructs in the model, which ensures the discriminant validity of the constructs.

Henseler et al. (2015) proposed the heterotrait–monotrait (HTMT) ratio of the correlations. Henseler et al. (2015) suggested 0.90 as a threshold value for structural models with dimensions. In this study, the values ranged from 0.100 to 0.746, which indicated that discriminate validity was established for all dimensions of the model, as shown in Table 4.

**TABLE 4:** Discriminant validity: Heterotrsait–monotrait (HTMT).

	1	2	3	4	5	6
(1) Online Purchase Intention						
(2) Enjoyment	0.493					
(3) Autonomy	0.360	0.306				
(4) Rewards	0.610	0.461	0.178			
(5) Absorption	0.452	0.584	0.059	0.373		
(6) Competition	0.528	0.485	0.100	0.746	0.528	

### Testing Structural Model Fit

Before proceeding to test the model, we first tested model fit by using three model fitting parameters: the standardized root mean square residual (SRMR), the normed fit index (NFI) and the exact model fit (bootstrap-based statistical inference). Henseler et al. (2015) introduced the SRMR as a goodness-of-fit measure for PLS-SEM that can be used to avoid model misspecification. NFI values above 0.9 usually represent acceptable fit. The third fit value is exact model fit, which tests the statistical (bootstrap-based) inference of the discrepancy between the empirical covariance matrix and the covariance matrix implied by the composite factor model. Dijkstra and Henseler et al. (2015) suggested the *d_LS* (i.e., the squared Euclidean distance) and the *d_G* (i.e., the geodesic distance) as two different ways to compute this discrepancy. A model fits well if the difference between the correlation matrix implied by the model being tested and the empirical correlation matrix is so small that it can be purely attributed to sampling error, thus the difference between the correlation matrix implied by your model and the empirical correlation matrix should be non-significant (*p* > 0.05). Henseler et al. (2015) considered that *d*_*ULS*_ and *d*_*G*_ are smaller than the 95% bootstrapped quantile (HI 95% of *d*_*ULS*_ and HI 95% of *d*_*G*_).

In this study, the SRMR value is 0.055 (<0.08) and the NFI is 0.912 (>0.90) and the *d*_*ULS*_ < bootstrapped HI 95% of *d*_*ULS*_ and *d*_*G*_ < bootstrapped HI 95% of *d*_*G*_, indicating the data fits the model well.

#### Inner Model Analysis

To assess the structural model, Hair et al. (2017) suggested looking at the R^2^, beta (β) and the corresponding t-values via a bootstrapping procedure with a resample of 5,000. They also suggested that in addition to these basic measures, researchers should also report the predictive relevance (Q^2^), as well as the effect sizes (f^2^). As asserted by Sullivan and Feinn (2012), while a *p*-Value can inform the reader whether an effect exists, it will not reveal the size of the effect. In reporting and interpreting studies, both the substantive significance (effect size) and statistical significance (*p*-Value) are essential results to be reported (p. 279). Hahn and Ang (2017) summarized some of the recommended rigor in reporting results in quantitative studies, which includes the use of replication studies, the use of effect size estimates and confidence intervals, the use of Bayesian methods, Bayes factors or likelihood ratios, and decision-theoretic modeling. Prior to hypotheses testing, the values of the variance inflation factor (VIF) have been determined. The VIF values are less than 5, ranging from 1.000 to 2.132. Thus, there have been no collinearity issues among the predictor latent variables (Hair et al., 2017).

**FIGURE 2:**
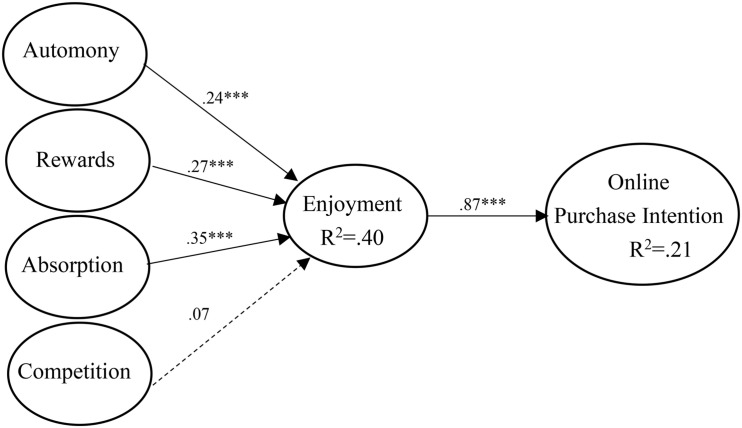
The results of PLS-SEM (*** if *p* < 0.001).

Figure 2 shows the test results of the structural model. The results in Table 5 show that reward has a positive impact on enjoyment (β = 0.27, *p* < 0.001); autonomy has a positive influence on enjoyment (β = 0.24, *p* < 0.001); absorption is positively correlated with enjoyment (β = 0.35, *p* < 0.001); and enjoyment has a positive correlation with purchase intention (β = 0.87, *p* < 0.001). Therefore, all hypotheses except for H5 have been supported. The Stone-Geisser Q^2^ values obtained through the blindfolding procedures for enjoyment (Q^2^ = 0.342) and online purchase intention (Q^2^ = 0.423) are larger than zero, confirming that the model has predictive relevance (Hair et al., 2017).

**TABLE 5:** Results of the paths.

Hypotheses	Std. β	*t*-value	Significance CI (2.50%-97.5%)	VIF	*f*^2^
H1: Enjoyment→ Online Purchase Intention	0.870***	8.349	(0.634∼0.934)	1.000	0.261
H2: Autonomy→ Enjoyment	0.240***	5.742	(0.149∼0.309)	1.032	0.084
H3: Rewards→ Enjoyment	0.270***	4.376	(0.114∼0.378)	1.892	0.012
H4: Absorption→ Enjoyment	0.350***	6.789	(0.273∼0.508)	1.316	0.191
H5: Competition→ Enjoyment	0.070***	1.166	(0.068∼0.309)	2.132	0.027

***If p < 0.001.

## Discussion and Conclusion

### Discussion

This study aims to explore how gamification affects consumer purchase intention. All the hypotheses except for H5 are supported, which provides powerful evidence for the model’s validity. This study shows that gamification can enhance consumer online purchase intention when game dynamics meets the psychological needs of consumers (Mullins and Sabherwal, 2020). It is worth noting that different game dynamics increase consumer satisfaction in different ways. Based on the results, this study proposes several specific contributions.

First, it is found that rewards, autonomy, and absorption of gamification elements enhance consumer enjoyment, and such consumer enjoyment promotes online purchase intention. This result is consistent with the importance of intrinsic motivation in the CET model as emphasized by other scholars (Deci and Ryan, 1980; Chae et al., 2017; Huotari and Hamari, 2017), according to which positive motivation and attitude of consumers can be produced via intrinsic motivation generated by gamification elements that enhance consumer enjoyment. The results also show that combining the above gamification factors satisfies the basic psychological needs of individuals, which is the key to enhancing the enjoyment in games, while the degree of enjoyment in games is the main determinant of consumer online purchase intention (Xi and Hamari, 2020). This also implies that the intrinsic motivation in CET is to be generated when people’s psychological needs are satisfied.

Moreover, the study results show that the enjoyment value in developing gamification within the online shopping market will promote consumer behavior. In prior marketing literature, some studies have employed CET to discuss consumer motivation and behavior (Webster and Ahuja, 2006; Sen et al., 2008; Seaborn and Fels, 2015); however, few studies have taken enjoyment as the important core intrinsic motivation, from the perspective of online marketing (Xi and Hamari, 2020), to induce consumers to have a specific consumer behavior, especially in relation to the gamification of online platform consumption. Although enjoyment value can enhance consumers’ online purchase intention, it also relies on important gamified antecedents, which is the element of game designing (Xi and Hamari, 2020). Games are generated when a group of different game elements are invoked by users in different environments. On this basis, we maintain that satisfaction of the basic psychological needs of consumers in the online shopping market is the key to the successful application of gamification. We also speculate that if any one of these psychological needs is ignored, the consumer enjoyment may be significantly reduced, and thus the consumer behavior may be adversely affected.

Finally, our study has found that competition has no positive effect on enjoyment. The competitive elements of a game may distract users and even lower their enjoyment (Lee and Yang, 2011). This result is similar to the argument that despite gamification comprising many game elements, not all these elements can successfully attract users (Hair et al., 1998; Huotari and Hamari, 2017). It would be impossible to attract consumers only by adding the enjoyment value through game elements without also considering how to meet the basic psychological needs of consumers. Previous studies have also held different views on the impact of competition, with some scholars suggesting that competition produces more driving force (Ryan and Deci, 2000a, b; Insley and Nunan, 2014; Mitchell et al., 2020). Other scholars have suggested that competition might have a negative effect on users’ psychological states when the competition is excessive or poorly designed such that it does not consider users’ characteristics (Qiu and Benbasat, 2010). The present study verifies that competition does not have a positive impact on consumer enjoyment in the online marketing context; however, our analysis also reveals a positive correlation between competition and consumer enjoyment, implying that well-designed competition in gamification motivates consumers in experiencing enjoyment (Mullins and Sabherwal, 2020).

In other words, the impact of each design element of gamification and the assessment of their impact on enjoyment are very important, and unreasonable design of competitive elements can reduce the degree of enjoyment.

### Implications for Research

This study makes important academic contributions. First, it extends CET by determining which antecedents among rewards, autonomy, and absorption can satisfy the need for enjoyment (Silverman, 2011; Santhanam et al., 2016; Huang et al., 2017). Researchers have found that CET can explain why people keep playing games (Deterding et al., 2011), but few studies have examined the impact of game-related factors on consumer online purchase intention (King et al., 2010). Our theoretical extensions help researchers develop their theories (Webster and Ahuja, 2006; Sen et al., 2008; Seaborn and Fels, 2015) and explain that some gamification elements are able to attract consumers and thus influence consumer behavior when people’s psychological needs are satisfied.

Secondly, this study explains four game elements that promote enjoyment and purchase intention. Our work shows that the design of gamification should be such that consumers satisfy their extrinsic and intrinsic regulation (autonomy, reward, and absorption) (Ryan and Deci, 2000a, b) and participate in the next action with the support of intrinsic motivation (Deci, 1985).

Thirdly, our conceptualization of structure and its measurement is beneficial for researchers as it enables them to more accurately monitor consumer behavior and analyze potential problems (Chen et al., 2015). In order to understand the impact of gamification on consumer purchase intention, researchers need to control and measure variables (Lee and Yang, 2011). To this end, and to make it more elaborate, the current work is conducive to the design of gamification.

### Implications for Practice

This study contributes to the extant literature on practice in the following ways. First, it can enlighten system designers and administrators who are trying to influence consumer behavior through gamification. Secondly, through this kind of research, practitioners or designers who are trying to improve the consumer experience can provide consumers with a higher level of enjoyment, thereby establishing a closer relation with consumers. Finally, as Kotler (1973) argued, a well-designed sales environment may have an emotional impact on consumers and increase the possibility of purchasing. Therefore, companies should create an environment that has a positive emotional impact on consumers.

The results of this study show that competition has no positive effect on enjoyment. Thus, the competitive dynamics that frequently occur in gamification design do not necessarily have a positive impact on motivating consumers. The competitive mechanism does not necessarily motivate consumers to enjoy the website more and increase their purchase intention. The model also contributes to the commercial application of gamification and provides relevant guidance for online shopping platforms in developing game designs and social cues; in addition, it contributes to future research in this new field.

This study also has a social significance. Many social media apps use reward and competition strategies that are common in games to make the utilization of apps more enjoyable for consumers (Silverman, 2011). Nevertheless, there is still a lack of prescriptive guidelines and design principles for successful application of gamification. The framework of this study has systematically explained how to help consumers enjoy themselves and make their online shopping more enjoyable. This, in turn, will pave the way for better gamified applications, and it will promote beneficial behaviors in the online society.

### Limitations and Further Research Directions

Although this study enables a better understanding of the impact of gamification on consumer online purchase intention, the impact of gamification on consumer enjoyment may change with variations in the design purposes of gamification systems. We appeal to researchers to study our model outside the field of the online shopping market, as there will be more developments and discoveries in research on gamification and consumer online purchase intention. For example, although we have found that competition has no positive effect on enjoyment, current studies have suggested that the impact of competition might vary according to skill levels and competitive structures (Liu et al., 2007). Therefore, in the future context of the development of gamification, further investigations are also required to be certain how different competitive structures affect enjoyment and online purchase intention.

The second limitation of this study is that our data may contain bias in its market selection. Because the object of this study is consumers participating in gamification on Taobao.com, such consumers may be more positive than those who are not attracted by gamification. Subsequent research could expand the research objects to people who are not sensitive to gamification.

We believe that our conceptualization of gamification and our empirical tests for consumer online purchase intention will lead to scholars paying more attention to gamification. We also emphasize that relevant theories need to be referred to as a basis before formulating effective gamification design strategies.

## Data Availability Statement

The raw data supporting the conclusions of this article will be made available by the authors, without undue reservation.

## Ethics Statement

The studies involving human participants were reviewed and approved by Institutional Review Board of National Kaohsiung University of Science and Technology. The patients/participants provided their written informed consent to participate in this study.

## Author Contributions

YX, ZC, and MP contributed to the ideas of educational research, collection of data, and empirical analysis. MP, ZC, MW, YP, and YX contributed to the data analysis, design of research methods, and tables. MP, MA, and YX participated in developing a research design, writing, and interpreting the analysis. All authors contributed to the literature review and conclusion, article, and approved the submitted version.

## Conflict of Interest

The authors declare that the research was conducted in the absence of any commercial or financial relationships that could be construed as a potential conflict of interest.

## Appendix

**TABLE A1:** Measurement items.

Construct	Measurement items	References	Factor loadings	*t*-value
Reward	When playing Stackopolis	Sen et al., 2008; O’Brien, 2010; Kankanhalli et al., 2012		
	(1) Awards increases my involvement in the game		0.94	25.40
	(2) I try to get more points as a reward for my activities.		0.93	77.29
	(3) I try to have a higher status as a reward for my activities.		0.94	84.83
	(4) I try to get more badges or loots as a reward for my activities.		0.82	101.88
Competition	When playing Stackopolis	Sheldon et al., 2001; Jang et al., 2009		
	(1) I am facing intense competition.		0.84	30.11
	(2) Activities of other participants are threats to me		0.85	31.93
	(3) Competition among participants is fierce.		0.85	28.67
	(4) Competition increases my participation in the game		0.83	50.14
Absorption	When playing Stackopolis	Seaborn and Fels, 2015		
	(1) I forget everything else around me.		0.88	46.82
	(2) Time flies.		0.87	41.42
	(3) I am immersed.		0.90	52.07
	(4) It is difficult to detach myself from the website.		0.89	43.52
Autonomy (AUT)	When playing Stackopolis	Sheldon et al., 2001; Jang et al., 2009		
	(1) I feel free to decide what to do for myself.		0.85	24.60
	(2) I feel that my choices are based on my true interests and values.		0.85	23.15
	(3) I feel free to do things on my own way.		0.90	42.12
Enjoyment (ENJ)	Stackopolis is	Kim et al., 2013		
	(1) interesting.		0.89	51.03
	(2) exciting.		0.93	97.29
	(3) fun.		0.82	35.28
Online Purchase intention	After the game,	Huang et al., 2017		
	(1) I bought goods or VIP services on the website.		0.87	36.42
	(2) I intend to pay for some goods or VIP services.		0.90	57.18
	(3) I am able to buy goods or VIP services with the acquired red packet.		0.81	31.54
	(4) I will recommend others to buy goods or VIP services.		0.67	12.33
